# Nrf2 protects against seawater drowning-induced acute lung injury via inhibiting ferroptosis

**DOI:** 10.1186/s12931-020-01500-2

**Published:** 2020-09-09

**Authors:** Yu-bao Qiu, Bin-bin Wan, Gang Liu, Ya-xian Wu, Dan Chen, Mu-dan Lu, Jun-liang Chen, Ren-qiang Yu, Dao-zhen Chen, Qing-feng Pang

**Affiliations:** 1 Wuxi School of Medicine, Jiangnan University, 1800 Lihu Avenue, Wuxi, 214122 Jiangsu Province People’s Republic of China; 2 Central Laboratory, The Affiliated Wuxi Maternity and Child Health Care Hospital of Nanjing Medical University, 48 Huaishu Lane, Wuxi, 214122 Jiangsu Province People’s Republic of China

**Keywords:** Acute lung injury, Ferroptosis, Nrf2, Drowning, Seawater

## Abstract

**Background:**

Ferroptosis is a new type of nonapoptotic cell death model that was closely related to reactive oxygen species (ROS) accumulation. Seawater drowning-induced acute lung injury (ALI) which is caused by severe oxidative stress injury, has been a major cause of accidental death worldwide. The latest evidences indicate nuclear factor (erythroid-derived 2)-like 2 (Nrf2) suppress ferroptosis and maintain cellular redox balance. Here, we test the hypothesis that activation of Nrf2 pathway attenuates seawater drowning-induced ALI via inhibiting ferroptosis.

**Methods:**

we performed studies using Nrf2-specific agonist (dimethyl fumarate), Nrf2 inhibitor (ML385), Nrf2-knockout mice and ferroptosis inhibitor (Ferrostatin-1) to investigate the potential roles of Nrf2 on seawater drowning-induced ALI and the underlying mechanisms.

**Results:**

Our data shows that Nrf2 activator dimethyl fumarate could increase cell viability, reduced the levels of intracellular ROS and lipid ROS, prevented glutathione depletion and lipid peroxide accumulation, increased *FTH1* and *GPX4* mRNA expression, and maintained mitochondrial membrane potential in MLE-12 cells. However, ML385 promoted cell death and lipid ROS production in MLE-12 cells. Furthermore, the lung injury became more aggravated in the Nrf2-knockout mice than that in WT mice after seawater drowning.

**Conclusions:**

These results suggested that Nrf2 can inhibit ferroptosis and therefore alleviate ALI induced by seawater drowning. The effectiveness of ferroptosis inhibition by Nrf2 provides a novel therapeutic target for seawater drowning-induced ALI.

## Background

Drowning is one of the main causes of accidental injury and death [1, 2]. It is estimated that more than 360,000 people die each year from drowning worldwide [3]. Moreover, it is worth noting that acute lung injury (ALI) is one of the most common complications of drowning and can develop ultimately into acute respiratory distress syndrome [4, 5]. Drowning damage alveolar epithelial cells, and thereafter cause hypoxia, hemorrhage and oxidative stress, and inflammation [6, 7]. Recent reports suggested oxidative stress played an important role in the pathogenesis drowning-induced ALI [4, 8]. Our previous studies have shown that heme oxygenase-1(HO-1) alleviated drowning-induced ALI [9]. Both several transcriptional factors (e.g. activator protein-1, hypoxia-inducible factor-1, nuclear factor-kappa B, nuclear factor erythroid 2-related factor-2 (Nrf2) and signaling cascades such as mitogen-activated protein kinase and phosphatidylinositol 3-kinase/Akt tightly regulated HO-1 gene expression [10]. Among these factors which can induce HO-1 expression, Nrf2 has a key role in regulating cellular antioxidant stress and eliminating reactive oxygen species (ROS) [11]. Recently research found that Nrf2 prevents oxidative stress-induced pulmonary diseases [12, 13]. However, whether or not Nrf2 is responsible for HO-1 induction in seawater-induced ALI is still unknown.

Ferroptosis is an iron-dependent, lipid peroxidation-driven cell death cascade and its main features are overwhelming cellular ROS production and iron-based lipid peroxide accumulation [14, 15]. Although the pathogenetic role of ferroptosis in ALI induced by seawater drowning is still elusive, its involvement in multiple diseases has been established. Nowadays, ferroptosis has been recognized as a key mechanism for cell death associated with ischemic organ injury, neurodegeneration and cancer [15–17]. Ferroptosis induced by GPX4 inhibition could prevent cancer resistant to chemotherapy [18, 19]. The latest research shows that inhibiting ferroptosis can alleviate ALI induced by lipopolysaccharide [20], cigarette smoke-induced chronic obstructive pulmonary disease [21] and radiation- induced lung fibrosis [22]. However, the role of ferroptosis in ALI induced by seawater drowning is unclear. In this study, we employed Nrf2 agonist dimethyl fumarate (DMF), Nrf2 inhibitor (ML385), Nrf2-knockout mice and ferroptosis inhibitor (Ferrostatin-1) to test the hypothesis that activation of the Nrf2 pathway attenuates drowning-induced ALI by inhibiting ferroptosis both in vivo and in vitro.

## Methods

### Reagents and antibodies

Artificial seawater (pH 8.2, osmolality 1300 mmol/L, specially weight 1.05, NaCl 26.518 g/L, MgSO4 3.305 g/L, MgCl_2_ 2.447 g/L, CaCl_2_ 1.141 g/L, KCl 0.725 g/L, NaHCO_3_ 0.202 g/L, and NaBr 0.083 g/L) was prepared according to the main components of seawater in the East China Sea provided by the China Oceanic Administration [23]. Dimethyl sulfoxide (DMSO) and dihydroethidium (DHE) were purchased from Sigma-Aldrich (St. Louis, MO, USA). Cell Counting Kit-8 (CCK-8) was purchased from Biosharp Life Sciences (Biosharp, China). DMF (Cat# HY-17363), ML385 (Cat# HY-100523) and Ferrostatin-1 (Fer-1, Cat# HY-100579) were obtained from Med Chem Express (MCE, USA). Primary antibodies for anti-GAPDH (Cat# ab181602) and anti-Nrf2 (Cat# ab62352) were obtained from Abcam (Abcam, USA).

DMF was suspended in 0.9% NaCl and 0.5% Methocel (Sigma) to a final concentration was 4 mg/ml. Fer-1 was dissolved in dimethyl sulphoxide and then diluted in saline to a final concentration of 0.5 mg/ml.

### Cell culture and treatment

MLE-12 cells (mouse lung epithelial cell line, obtained from ATCC, USA) were maintained in DMEM (GIBCO, USA) medium supplemented with 10% fetal bovine serum (FBS, GIBCO, USA) and 1% penicillin-streptomycin solution (GIBCO, USA) and placed in a cell culture chamber containing 5% CO_2_ at 37 °C. MLE-12 cells were seeded in 96-well plates at a concentration of 5 × 10^4^/ml, or in 6-well plates at a concentration of 1 × 10^5^/ml, and used for experiments after overnight cultivation.

MLE-12 cells were treated with a final concentration of 25% seawater (0.25 ml per 1 mL total volume) for 2, 4, 6, 8 or 12 h, respectively. At the same time, the cells inoculated in complete medium and cultured without seawater and drugs were used as the control group. Finally, the treatment was ended and tested at the same time to explore the time dependence of the effect of seawater on MLE-12 cells. In subsequent experiments, cells exposed to 25% seawater for 6 h were used as the seawater (SW) group [9]. The other drug treatment groups were pretreated with final concentrations of Fer-1 (10 μM), DMF (20 μM) and ML385 (20 μM) for 2 h before seawater exposure [24–26].

### Cell viability assay

Cells viability was measured by using Cell Counting Kit-8 (CCK-8) kits. Cells were seeded at a density of 1 × 10^4^/well in 96-well plates. After washing the cells with phosphate buffered saline (PBS), the CCK-8 solution was added to the medium at a dilution of 1:10 and incubated at 37 °C for 2 h. Absorbance was measured at 450 nm using a microplate reader (BioTek, Winooski, USA).

### Determination of the activities of superoxide dismutase (SOD), the content of glutathione (GSH) and malondialdehyde (MDA)

The activities of SOD and the contents of GSH and MDA in MLE-12 cells and lung tissues were measured by a commercial assay kit (Nanjing Jiancheng Bio Co., Ltd., China) according to the manufacturer’s instructions.

### Detection of intracellular ROS by fluorometric intracellular ROS kit

The level of intracellular ROS was measured using a fluorometric ROS kit (Nanjing Jiancheng Bio Co., Ltd., China). MLE-12 cells was incubated with 10 μM 2′,7′-dichlorodihydrofluorescein diacetate (DCFH-DA) in the dark for 1 h and then washed with PBS. The fluorescence intensity was measured by a fluorescence spectrophotometer (wavelength was 485 nm and the emission wavelength was 530 nm).

### Intracellular ROS concentrations using DHE fluorescent probe

MLE-12 cells were treated with 10 μM dihydroethidium (DHE) in the dark for 30 min and then washed with PBS. Images were observed and collected by a Nikon TE-2000 fluorescence microscope (Nikon, Tokyo, Japan).

### Mitochondrial membrane potential assay

Mitochondrial membrane potential (MMP) of MLE-12 cells were detected using fluorescent probe JC-1 and rhodamine 123 (Sigma-Aldrich, St Louis, MO). Briefly, cells were incubated for 30 min at 37 °C in the dark with JC-1 (5 μM) or rhodamine 123 (5 μM). After washing with PBS, cell fluorescence images by JC-1 staining were observed and obtained using a Nikon TE-2000 fluorescence microscope (Nikon, Tokyo, Japan). The fluorescence intensity of rhodamine 123 staining was measured with a microplate reader using a 485 nm excitation and a 529 nm emission filter setup.

### Collection of mitochondrial superoxide images by MitoSox red fluorescent probe

MLE-12 cells were treated with 5 μM MitoSox Red (Invitrogen) in the dark for 30 min and then washed with PBS. Images were observed and collected by a Nikon TE-2000 fluorescence microscope (Nikon, Tokyo, Japan).

### Flow cytometric analysis of cell death and lipid ROS

Cell death was measured by flow cytometry (BD Biosciences, C6 Plus, USA) using Annexin V-FITC/PI Apoptosis Detection Kit (Beijing Cowin Biotech Co., Ltd., China) according to the manufacturer’s instructions [17]. The assay for lipid ROS was performed by incubating the cells for 1 h at a final concentration of 2 μM of BODIPY 581/591 C11 (Invitrogen) at 37 °C. The cells were then washed twice with PBS, resuspended in PBS, and analyzed by flow cytometry (BD Biosciences, C6 Plus, USA) [17, 27].

### Seawater drowning model and treatments

Eight-week-old Nrf2-knockout (Nrf2^−/−^) and wild-type (WT) littermate male mice on a C57BL/6 J background (obtained from Model Animal Research Center, MARC, Nanjing, project No. XM002783) were used to conduct the in vivo experiments. For the mice drowning model, mice were placed in a porous container and immersed in a water bath containing 6 cm deep 25 ± 2 °C for 35 s [9, 28]. All experiments were conducted in accordance with established guidelines and approved by the Animal Care and Use Committee of Jiangnan University (JN. No. 20180615b0841230). Wild-type mice were randomly assigned to 6 groups: control, SW, Fer-1, SW + Fer-1, DMF and SW + DMF. Nrf2-knockout mice were randomly assigned to 2 groups: Nrf2 KO and Nrf2 KO + SW.

DMF group and SW + DMF group were given DMF (80 mg/kg) by gavage at 3 h, 24 h and 48 h after drowning, with a volume of 0.4–0.5 ml per mouse [29]. Fer-1 was diluted with 0.01% DMSO in saline to a final concentration of 0.5 mg/ml. Fer-1 and SW + Fer-1 groups were given Fer-1 (5 mg/kg) by intraperitoneal injection at 3 h, 24 h and 48 h after drowning, with a volume of 0.2–0.25 ml per mouse [30]. All groups were sacrificed on the third day after drowning and lung tissue samples were collected for evaluation.

### Tissue collection, wet-to-dry ratio analysis and histological analysis

Right lungs were collected for determined the lung wet-to-dry ratio, western blot and biochemical analysis. The lungs were dissected, weighed, and dried in an oven at 60 °C for 72 h, and then the wet-to-dry ratio was calculated to evaluate tissue edema. The left lung tissue sample was filled with 4% paraformaldehyde by tracheal infusion, and then placed in 4% paraformaldehyde solution for fixation. After the fixation, the left lung tissue samples were embedded in paraffin. Tissue pieces were cut into 4 μm sections and stained with hematoxylin and eosin (HE) for lung injury score [31].

### Micro-computed tomographic analysis

Mice were anesthetized with isoflurane continuously delivered through a nose cone and scanned using a Quantum FX micro-CT Imaging System (PerkinElmer, USA). Each mouse was scanned for 4 min under the parameters of 70 kV, 88 μA, 36 mm FOV. The data acquired by the scan was analyzed and 3D reconstructed using the Analyze 12.0 software at the same level setting.

### Immunofluorescence staining of Nrf2 in vitro and in vivo

To observe Nrf2 localization of MLE-12 cells. Cells were then fixed in 4% formaldehyde and permeabilized with 0.1%Triton X. The cells were probed with Nrf2 antibodies followed by Alexa Fluor 488-conjugated secondary antibodies (Thermo Fisher Scientific, USA). To visualize the nuclei, cells were then treated with 1 μg/ml DAPI for 10 min and then washed by PBS. Finally, added the anti-fade mounting medium and collected images using Zeiss LSM 880 laser confocal fluorescence microscope (Carl Zeiss, Oberkochen, Germany).

To observe the expression of Nrf2 in mouse lung tissue. Paraffin slides were rehydrated in alcohol with decreasing concentrations and then placed in 10 mM sodium citrate buffer heated to 95 °C for 30 min for antigen retrieval. Then, the slides were permeabilized with 0.5% Triton-X and blocked with 5% BSA for 2 h. The slides were probed with Nrf2 antibodies followed by Alexa Fluor 555-conjugated secondary antibodies (Thermo Fisher Scientific, USA). The slides were incubated in DAPI for 10 min and then washed. Finally, the slides were mounted with an anti-fade mounting medium and collected images using Zeiss Axio Imager 2 fluorescence microscope (Carl Zeiss, Oberkochen, Germany).

### Western blot

The samples were homogenized in ice-cold RIPA buffer with protease inhibitor mixture, and the supernatant was collected after centrifugation (12,000 rpm, 10 min and 4 °C). The protein concentrations were measured using a BCA kit, and proteins were then denatured at 100 °C for 5 min. Proteins were loaded onto a 10% SDS-PAGE gel and transferred to a nitrocellulose filter membrane. Blots were blocked at room temperature for 1 h and then incubated with primary antibodies at 4 °C overnight. After blocking and washing, the blot was incubated with the secondary antibody for 2 h. Protein bands were visualized and analyzed by the ChemiDocTM XRS Plus luminescence image analyzer (Bio-Rad, USA) using the ECL system (Millipore, USA).

### Reverse transcription-quantitative polymerase chain reaction

Total RNA isolation and quantitative real-time PCR were performed using the procedure described previously [32]. The primers used in this study were synthesized by GENEWIZ Biotechnology Co., Ltd. (Suzhou, China) (Table 1).

**Table 1:** Primers used in this study for PCR

Gene	Forward	Reverse
*Nrf2*	5′-AAAATCATTAACCTCCCTGTTGAT-3′	5′-CGGCGACTTTATTCTTACCTCTC-3′
*Ptgs2*	5′-AAGTGCGATTGTACCCGGAC-3′	5′-GTGCACTGTGTTTGGAGTGG-3′
*GPX4*	5′-CTTATCCAGGCAGACCATGTGC-3′	5′-CCTCTGCTGCAAGAGCCTCCC-3′
*FTH1*	5′-GCACACTCCATTGCATTCAGCC-3′	5′-GCCGAGAAACTGATGAAGCTGC-3′
*GAPDH*	5′-TGTGATGGGTGTGAACCACGAGAA-3′	5′-GAGCCCTTCCACAATGCCAAAGTT-3′

### Protein–protein interaction analysis

Search Tool for the Retrieval of Reciprocity Genes (STRING) online database (http://string-db.org/) was used to evaluate protein–protein interaction network of Nrf2, Ptgs2, GPX4 and FTH1. Organism was selected “*Mus musculus*”. The online database provided assessment and integration of protein interactions, including direct (physical) and indirect (functional) correlations [33].

### Statistical analysis

Measurement data were expressed as means ± standard deviation (SD). Comparisons among groups were carried out by analysis of variance (ANOVA) with GraphPad Prism. *P* < 0.05 was considered statistically significant.

## Results

### Seawater exposure induced ferroptosis in MLE-12 cells

**Fig. 1:**
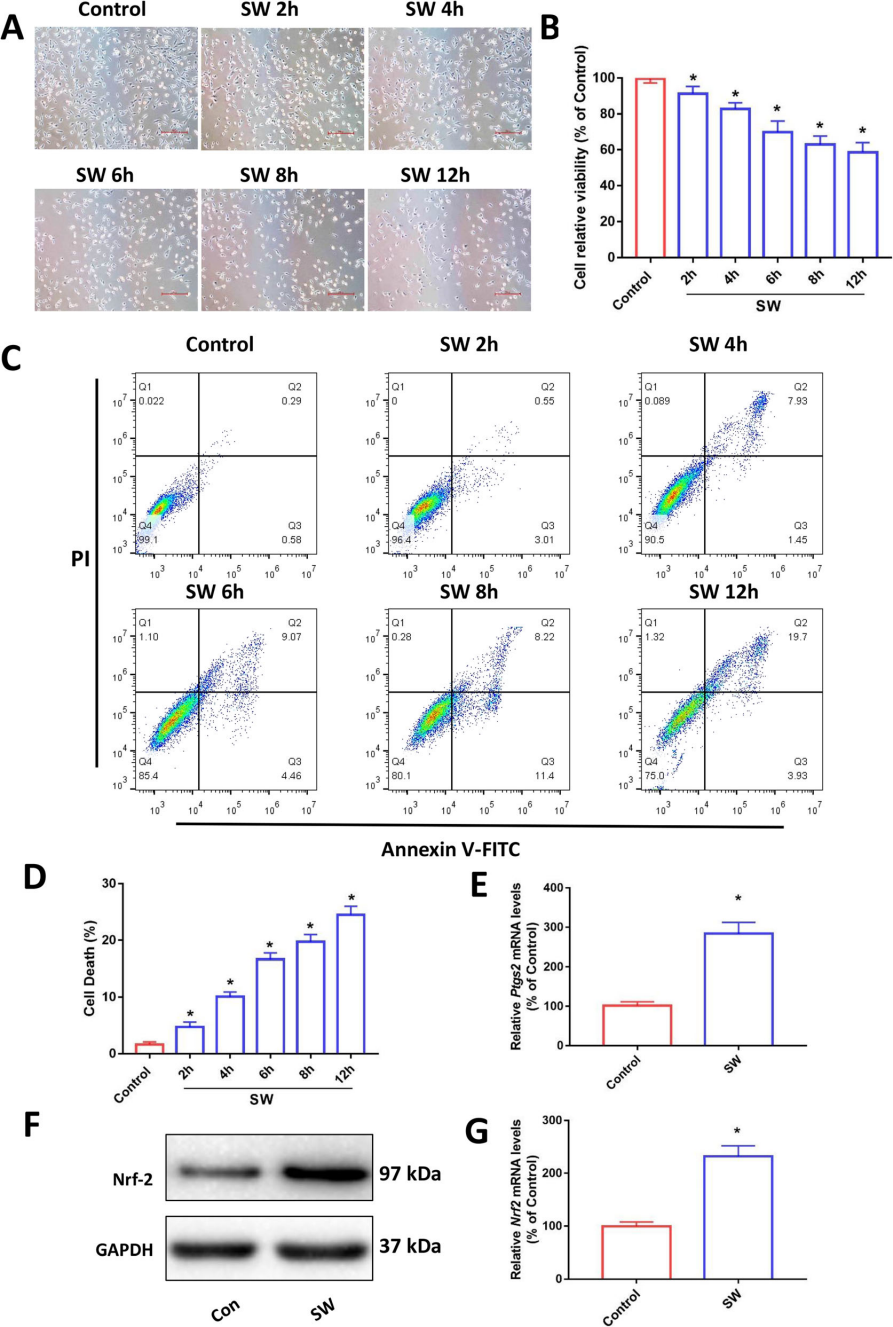
Seawater exposure induced MLE-12 cell damage. MLE-12 cells were exposed to 25% seawater for different times (**a**-**d**). (**a)** Cell morphology images (Original magnification × 200. Bar = 50 μm). (**b**) CCK-8 assay for the determination of cell viability (*n* = 4). (**c**) Cell death was analyzed using Annexin V-FITC/PI staining with flow cytometry (*n*=3). (**d**) Quantitative results of the percentage of cell death (*n* = 3). (**e**) The expression of Ptgs2 mRNA in MLE-12 cells was measured by qPCR after 6 h of seawater exposure (*n* = 4). The level of Nrf2 expression was determined by Western blot (**f**) and qPCR (**g**) (*n* = 3). Data were presented as the mean ± SD. ^⁎^*P* < 0.05 vs. Control group

In order to explore the time correlation of the effect of seawater on MLE-12 cells. MLE-12 cells were treated with seawater at a final concentration of 25% for 2, 4, 6, 8 or 12 h, respectively, and the treatment was ended and tested at the same time. We observe the cells by bright field microscope and use CCK8 kit to detect cell viability. The results showed that seawater exposure changed MLE-12 morphology (Fig. 1a) and decreased cellular viability in time-dependent manner (Fig. 1b). The results of flow cytometry detection of death also showed an increase in the proportion of cell death as the seawater exposure time prolonged (Fig. 1c and d) [17]. These results indicate that seawater exposure caused MLE-12 cell damage and death. Based on the above experiments, this study selected seawater stimulation for 6 h as the follow-up test conditions. As shown in Fig. 1e, seawater stimulation induced a significant increase in *Ptgs2* mRNA in MLE-12 cells, a key molecular marker of ferroptosis [19, 34]. Both *Nrf2* mRNA and protein expression in MLE-12 cells also increased after 6 h of seawater stimulation (Fig. 1f and g).

**Fig. 2:**
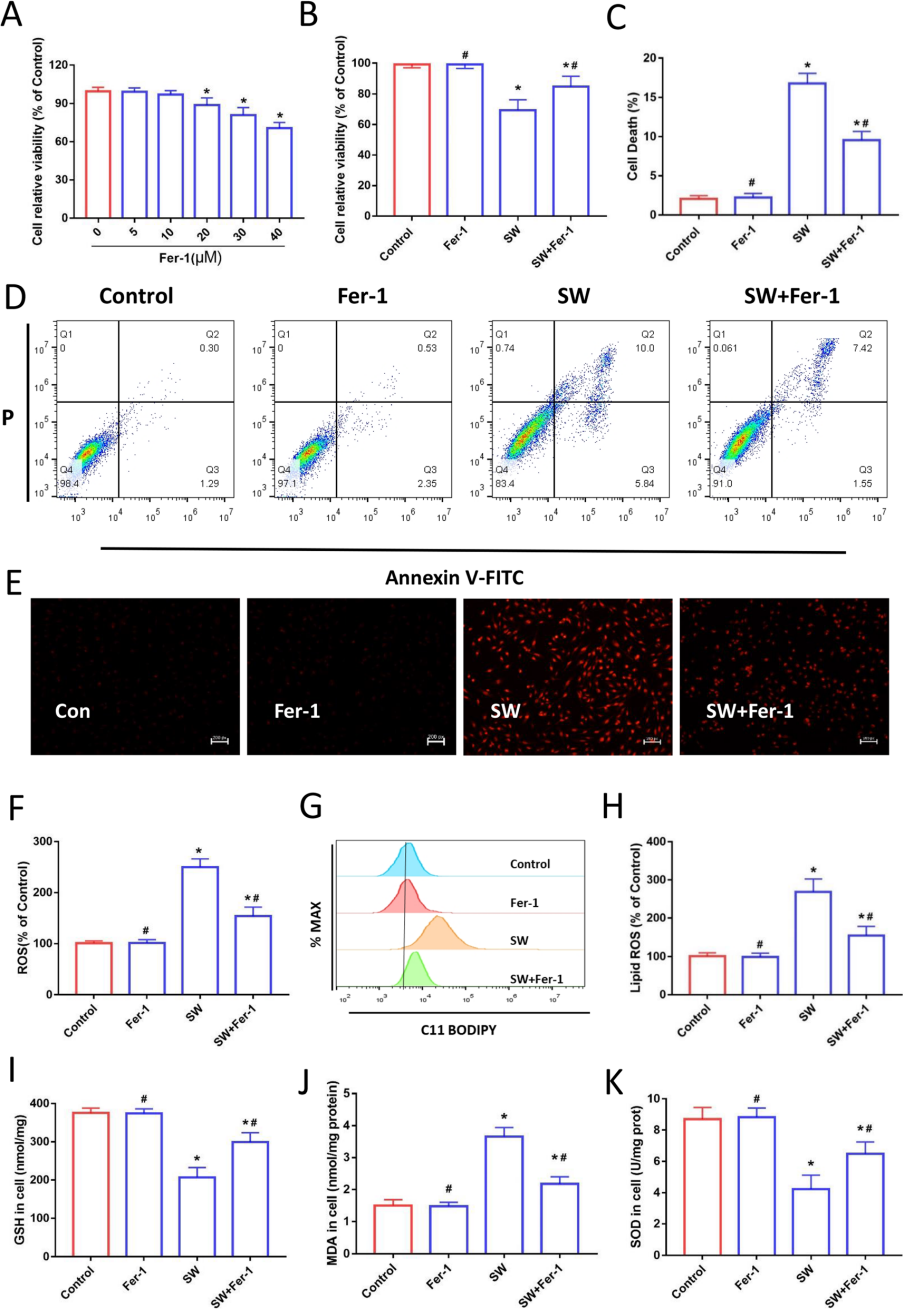
Inhibition of ferroptosis alleviated seawater-induced MLE-12 cell damage. (**a**) and (**b**). CCK-8 assay for the determination of cell viability (*n* = 4). (**d**) Cell death was analyzed using Annexin V-FITC/PI staining with flow cytometry (*n* = 3). (**c**) Quantitative results of the percentage of cell death (*n* = 3). (**e**) Intracellular ROS was monitored using the DCFH-DA fluorescent probes (*n* = 4). (**f**) Images of intracellular ROS levels in MLE-12 cells stained by DHE (10 μM) fluorescent probes. Scale bars: 50 μm. (**g**) lipid ROS was analyzed using 2 μM BODIPY® 581/591 C11 with flow cytometry (*n* = 3). (**h**) Quantitative results of lipid ROS (*n* = 3). Kit detected GSH (**i**), MDA (**j**) and SOD (**k**) levels in MLE-12 cells (*n* = 4). Data were presented as the mean ± SD. ^⁎^*P* < 0.05 vs. Control group, ^#^*P* < 0.05 vs. SW group

To investigate whether ferroptosis participated in MLE-12 cell death induced by seawater drowning, we pretreated cells with the ferroptosis specific inhibitor Fer-1. MLE-12 cells were treated with Fer-1 at a final concentration of 5, 10, 20, 30 and 40 μM for 24 h (Fig. 2a). Because the concentration of 10 μM Fer-1 did not significantly influence cell viability, 10 μM Fer-1 was used as the concentration for subsequent experiments [26]. As shown in Fig. 2b-d, the percentage of cell death after Fer-1 treatment was reduced and cell viability was increased in the SW + Fer-1 group relative to the SW group. The fluorescent indicator (DCFH-DA) and dihydroethidium (DHE) fluorescent probes were used to monitor the generation of intracellular ROS in seawater-treated MLE-12 cells. The two detection methods consistently showed that seawater exposure caused a significant increase of intracellular ROS in MLE-12 cells, which was decreased by Fer-1 treatment (Fig. 2e and f). Lipid ROS accumulation is a typical feature of ferroptosis [35]. The results of flow cytometry after C11-BODIPY®581/591 staining showed that lipid ROS levels were significantly reduced after Fer-1 treatment compared with SW group (Fig. 2g and h). Glutathione depletion and lipid peroxide accumulation are important features of ferroptosis [36]. MDA is the most common by-product of lipid peroxidation [37]. Fer-1 treatment reversed the decrease of GSH level and SOD activity caused by seawater stimulation (Fig. 2i and k). Meanwhile, Fer-1 also significantly reduced the content of MDA (Fig. 2j). These results suggest that ferroptosis is involved in MLE-12 cell damage induced by seawater stimulation.

### Nrf2 activation attenuates seawater-induced MLE-12 cell damage

**Fig. 3:**
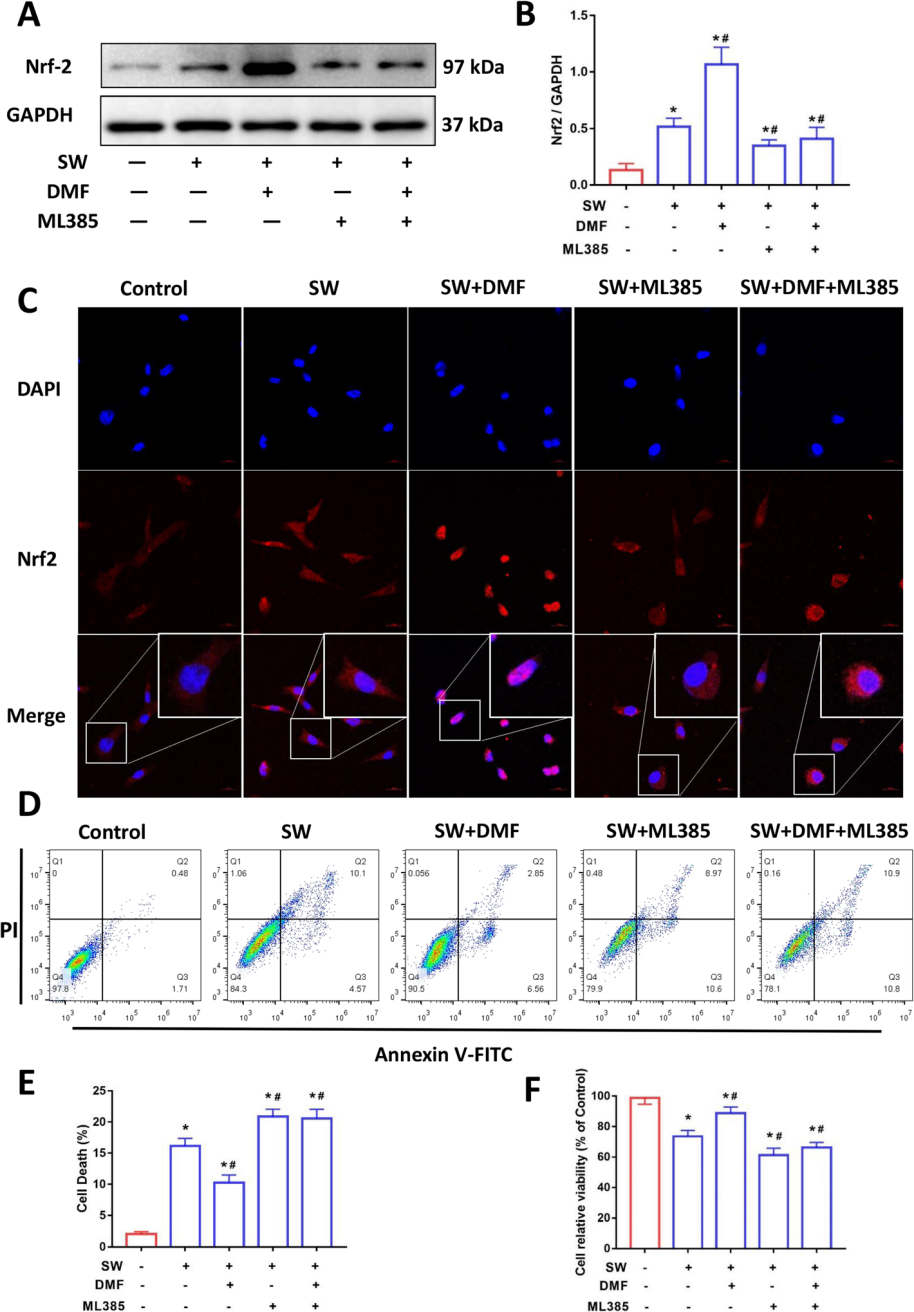
Nrf2 attenuated seawater-induced MLE-12 cell damage. Images (**a**) and quantification (**b**) of Nrf2 expression levels were analyzed by Western blot (*n* = 3). (**c**) Nrf2 was detected by immunofluorescence (Red: Nrf2, Blue: nucleus, Original magnification × 400. Bar = 20 μm). (**d**) Cell death was analyzed using Annexin V-FITC/PI staining with flow cytometry (*n* = 4). (**e**) Quantitative results of the percentage of cell death (*n* = 4). (**f**) CCK-8 assay for the determination of cell viability (*n* = 4). Data were presented as the mean ± SD. ^⁎^*P* < 0.05 vs. Control group, ^#^*P* < 0.05 vs. SW group

To investigate the effect of Nrf2 on MLE-12 cell damage induced by seawater stimulation, we treated cells with DMF (20 μM) and/or ML385 (20 μM). As shown in Fig. 3a and b, DMF did significantly increase the expression of Nrf2 whereas ML385 treatment reduced the expression level of Nrf2. As a transcription factor, translocation into the nucleus is a key step for Nrf2 to play a regulatory role. Immunofluorescence images also showed that DMF significantly promoted the translocation of Nrf2 into the nucleus, while ML385 suppressed this phenomenon (Fig. 3c). Flow cytometry results showed that DMF reduced the percentage of cell death caused by seawater stimulation, whereas ML385 reversed this effect (Fig. 3d and e). The results of CCK8 also showed that compared with the SW group, the cell viability significantly increased in the SW + DMF group, while the cell viability of the SW + ML385 group and the SW + DMF + ML385 group decreased (Fig. 3f). These results indicated that Nrf2 activation could attenuate cell death induced by seawater stimulation in MLE-12 cells.

### Nrf2 acivation inhibited seawater-induced ferroptosis in MLE-12 cells

**Fig. 4:**
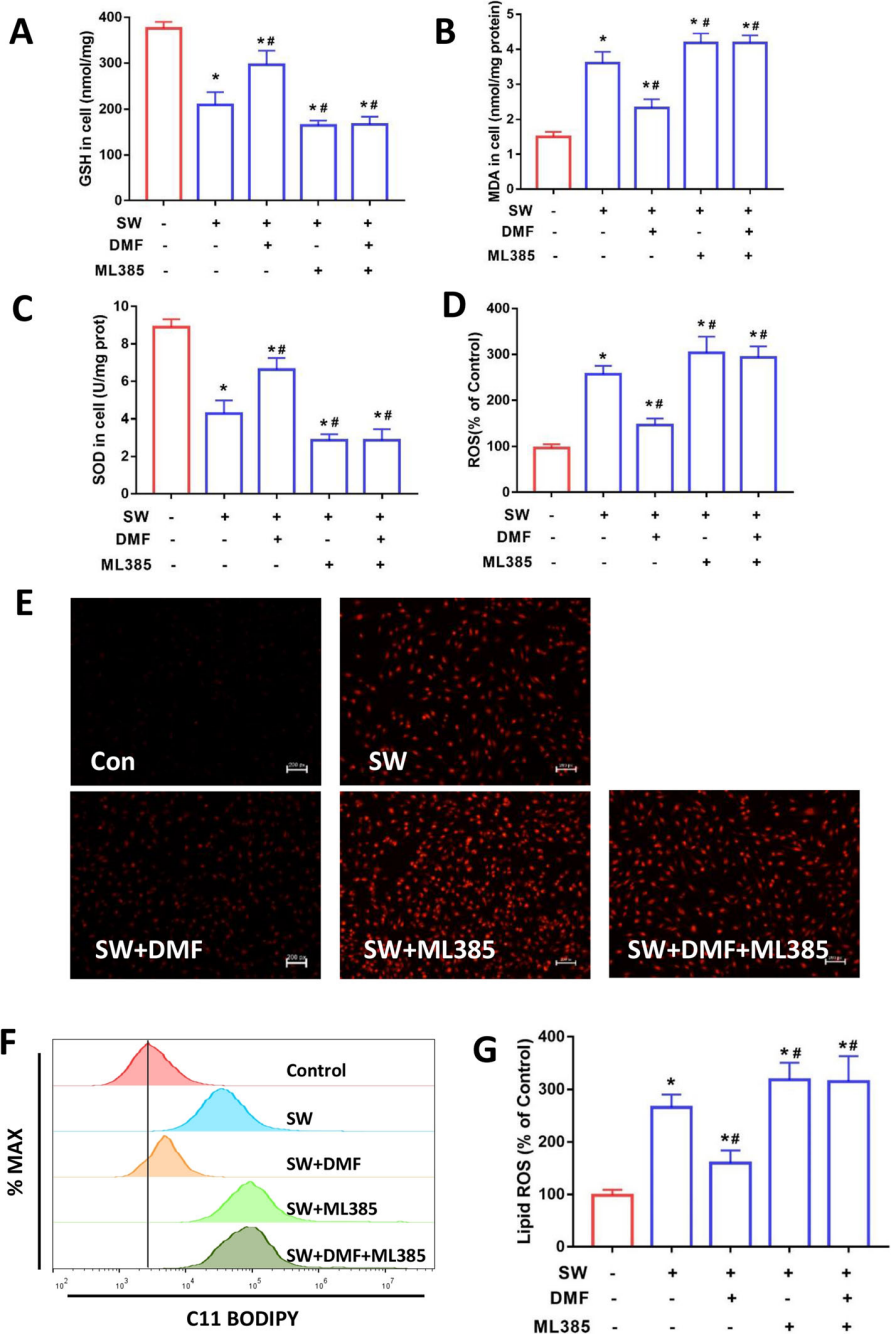
Nrf2 alleviated GSH depletion, lipid peroxidation and accumulation of ROS induced by seawater stimulation in MLE-12 cells. The content of GSH (**a**), MDA (**b**) and SOD (**c**) in MLE-12 cells (*n* = 4). (**d**) Intracellular ROS was monitored using the DCFH-DA fluorescent probes (*n* = 4). (**e**) Images of intracellular ROS levels in MLE-12 cells stained by DHE (10 μM) fluorescent probes. Original magnification × 200. Bar = 50 μm. (**f**) lipid ROS was analyzed using 2 μM BODIPY® 581/591 C11 with flow cytometry (*n* = 3). (**g**) Quantitative results of lipid ROS (*n* = 3). Data were presented as the mean ± SD. ^⁎^*P* < 0.05 vs. Control group, ^#^*P* < 0.05 vs. SW group

We further explored the role of Nrf2 on seawater-induced ferroptosis in MLE-12 cells. As shown in Fig. 4a-c, DMF significantly increased the GSH content and SOD activities and decreased the content of MDA, whereas ML385 reversed these effects. Furthermore, the detection of intracellular ROS by the fluorescent dye DCFH-DA showed that seawater-induced ROS levels were reduced after DMF treatment, while ML385 increased ROS levels (Fig. 4d). The same results were obtained for the fluorescent images of DHE staining (Fig. 4e). The results of flow cytometry for detecting lipid ROS also indicated that ML385 abolished the effect of DMF on reducing lipid ROS accumulation induced by seawater stimulation (Fig. 4f and g).

**Fig. 5:**
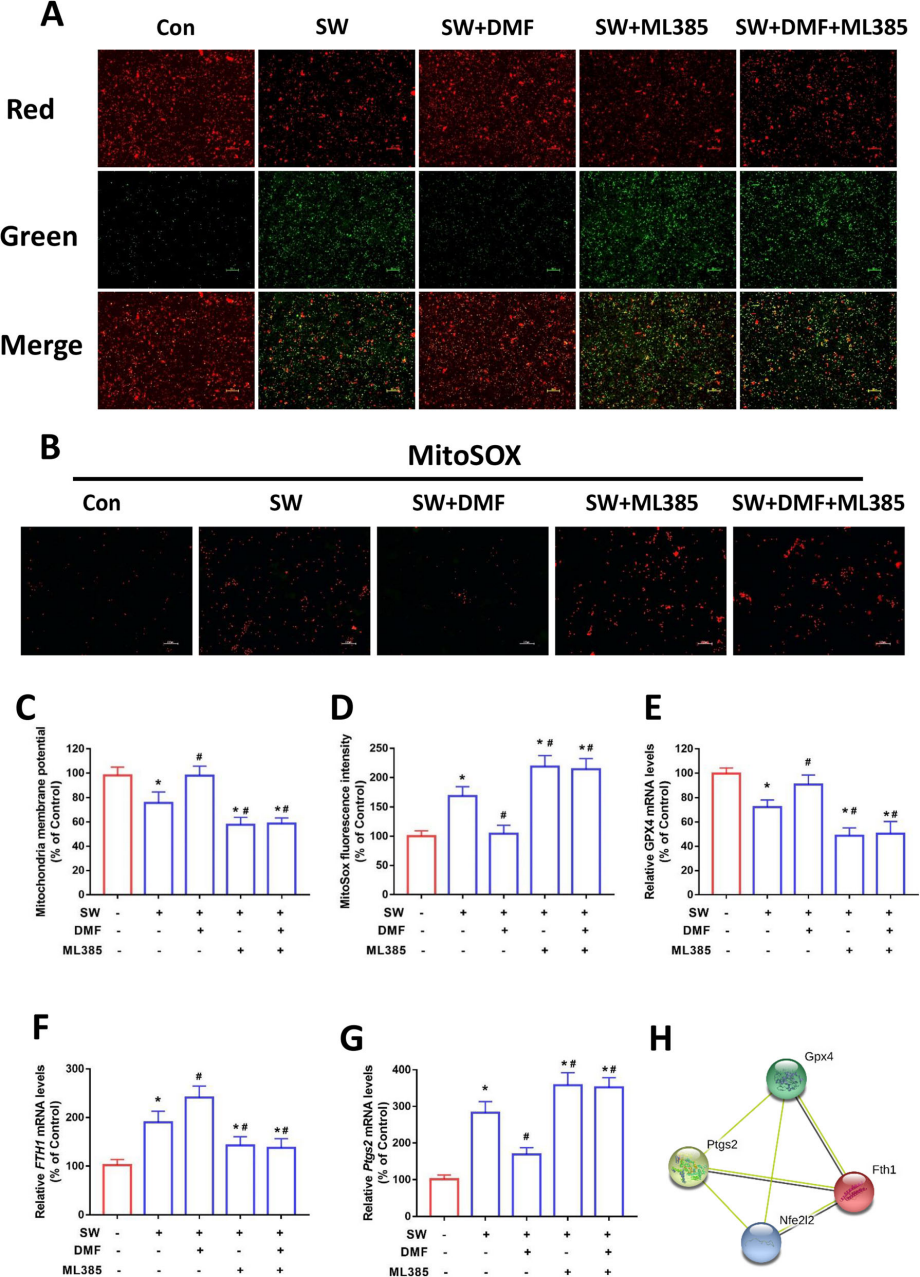
Nrf2 protected mitochondria and regulated ferroptosis related gene expression. (**a**) Fluorescence images of 5 μM JC-1 stained MLE-12 cells. Scale bars: 50 μm. The ratio of red and green fluorescence reflected changes of mitochondrial membrane potential (*n* = 3). Fluorescence images (**b**) and relative quantification (**d**) of MitoSox Red (5 μM) stained MLE-12 cells. Original magnification × 200. Bar = 50 μm. (**c**) The mitochondrial membrane potential stained with rhodamine 123 (5 μM) was detected by a microplate reader (*n* = 4). Relative mRNA expression of ferroptosis-related genes GPX4 (**e**), FTH1 (**f**) and Ptgs2 (**g**) in MLE-12 cells (*n* = 4). (H) The protein–protein interaction (PPI) network of Nrf2, GPX4, Ptgs2 and FTH1 (String). Data were presented as the mean ± SD. ^⁎^*P* < 0.05 vs. Control group, ^#^*P* < 0.05 vs. SW group

Mitochondria are important organelles of oxidative metabolism and play a crucial role in ferroptosis [38]. Mitochondrial membrane potential (MMP) was detected by JC-1 staining fluorescence image and rhodamine 123. As shown in Fig. 5a and c, DMF has a protective effect on seawater-induced mitochondrial membrane potential reduction (mitochondrial depolarization), while ML385 reverses this effect. MitoSox Red is a mitochondria-targeted ROS dye. Fluorescence images and intensity results indicate that ML385 reverses the effect of DMF on decreasing mitochondrial ROS levels (Fig. 5b and d). GPXs can use the reduced GSH to eliminate peroxides, of which glutathione peroxidase 4 (GPX4) is the main neutralizer of lipid peroxides [16]. We found that DMF treatment up-regulated *GPX4* mRNA levels in MLE-12 cells after seawater exposure, whereas ML385 caused a decrease in *GPX4* mRNA (Fig. 5e). FTH1 is an important gene for storing iron and maintaining iron homeostasis [39, 40]. As shown in Fig. 5f, DMF promoted the expression of *FTH1* mRNA, whereas ML385 reversed this effect. As a potential marker of ferroptosis, Ptgs2 also showed a significant decrease in mRNA level relative to SW group after DMF treatment, while the levels of SW + ML385 group and SW + DMF + ML385 group were further increased (Fig. 5g). A network of Ptgs2, GPX4 and FTH1 that significantly interacted with Nrf2 was constructed using the String database (protein–protein interaction). The network graphic showed that ferroptosis-related genes *FTH1*, *GPX4* and *Ptgs2* were clearly associated with Nrf2 (Fig. 5h). The results of the above various tests indicated that DMF promoted Nrf2 expression and inhibited ferroptosis in the MLE-12 cell after seawater exposure, and this beneficial effect was abolished by the Nrf2 inhibitor ML385.

### Fer-1 ameliorated lung injury induced by seawater drowning in mice

**Fig. 6:**
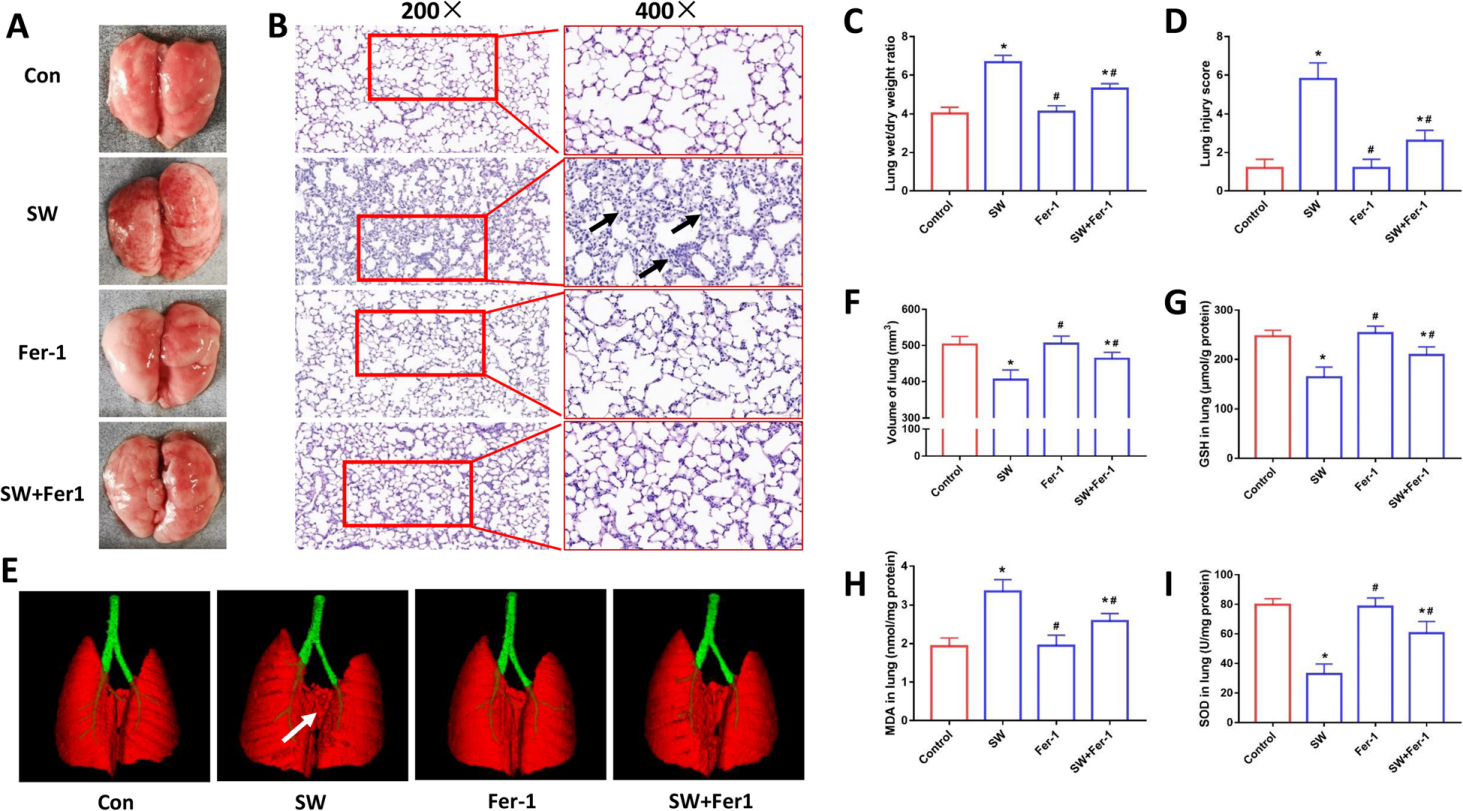
Inhibition of ferroptosis ameliorated lung injury in mice induced by seawater drowning. (**a**) Gross morphology of mice lung. H&E stained pathological tissue images (**b**) and ALI scores (**d**) (*n* = 4). Original magnification × 200 and × 400. Bar = 50 μm. (**c**) Lung wet/dry weight ratio (*n* = 4). (**e**) Micro CT images of mouse lungs. (**f**) Lung volume quantification was analyzed by Analyze 12.0 software based on the Micro CT data (*n* = 4). Kit detected GSH (**g**), MDA (H) and SOD (**i**) levels in mouse lung tissue (*n* = 4). Data were presented as the mean ± SD. Values are mean ± SD of four experiments. ^⁎^*P* < 0.05 vs. Control group, ^#^*P* < 0.05 vs. SW group

To further validate the above findings, we performed in vivo experiments by using a mouse drowning lung injury model. As shown in Fig. 6a, obvious edema and hemorrhage occurred in the gross anatomy of the lung on the third day after seawater drowning while the ferroptosis inhibitor (Fer-1) treatment improved this condition. When compared that in the SW group, the SW + Fer-1 group showed a significant decrease in lung wet-to-dry ratio, which indicated a reduction in pulmonary edema (Fig. 6c). H&E-stained sections showed that Fer-1 attenuated lung injury caused by seawater drowning (Fig. 6b and d). We further tested mouse lung injury by Micro CT. The lung volume of the mice was calculated by the Analyze 12.0 software based on the Micro CT data. As shown in Fig. 6e, seawater drowning caused obvious damage and deformation of the mouse lung, but Fer-1 treatment ameliorates this pathological change. Compared with the SW group, the lung volume of the SW + Fer-1 group also recovered significantly (Fig. 6f). We also tested the content of GSH and MDA and SOD activity in lung tissue, and the results showed that Fer-1 treatment improved the reduction of GSH content and SOD activities and decreased MDA levels (Fig. 6g-i). These results indicated that inhibition of ferroptosis could ameliorate lung injury in mice caused by seawater drowning.

### DMF inhibited ferroptosis and attenuated lung injury induced by seawater drowning in mice

**Fig. 7:**
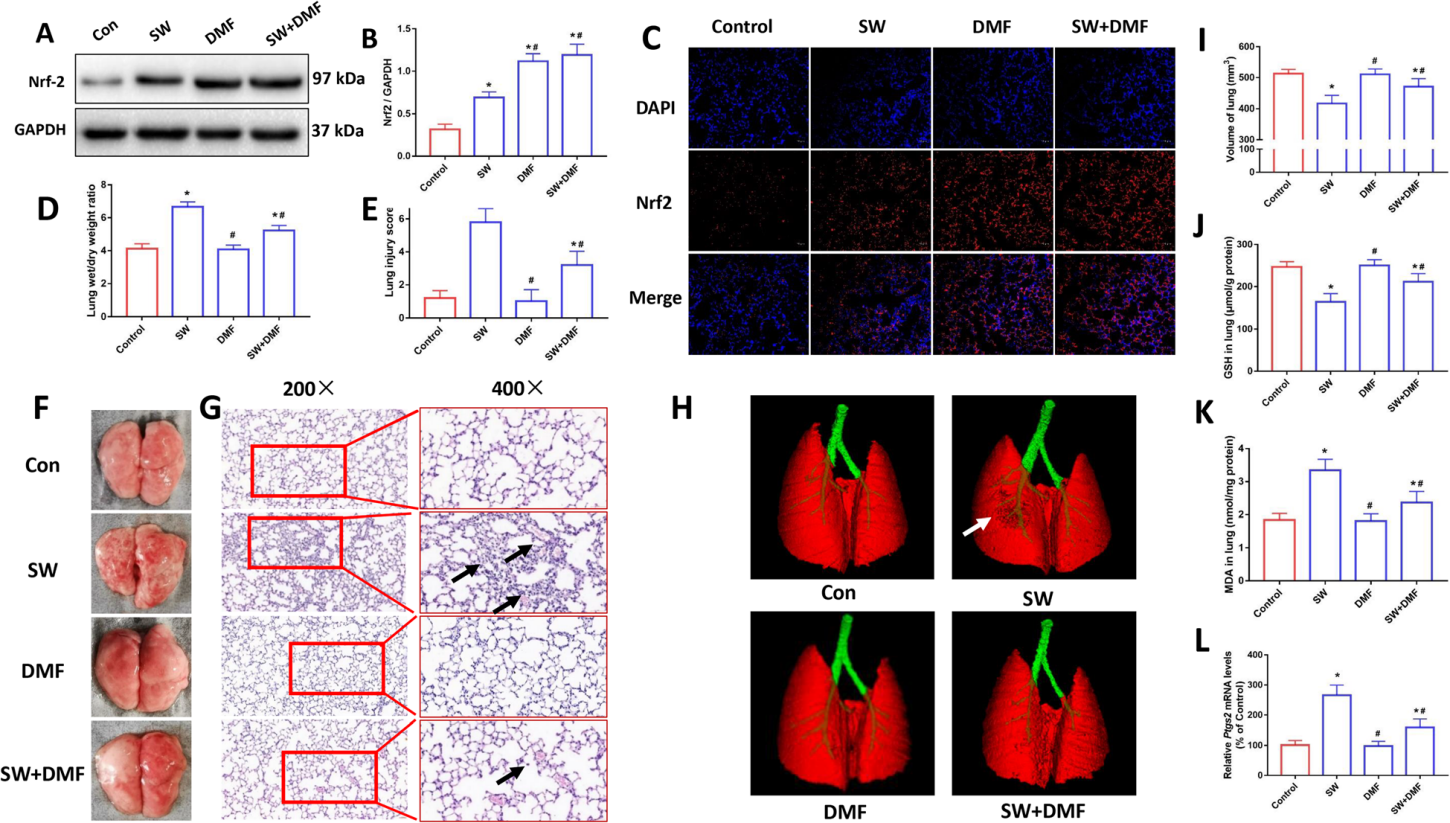
Nrf2 agonist DMF inhibited ferroptosis and improved ALI in mice induced by seawater drowning. Images (**a**) and quantification (**b**) of Nrf2 expression level in lung were analyzed by Western blot (*n* = 3). (**c**) Immunofluorescence images were used to detect the expression of Nrf2 (red) in mouse lung tissue (*n* = 3). Nuclei were counterstained with DAPI (blue). Original magnification × 200. Bar = 50 μm. (**d**) Lung wet/dry weight ratio (*n* = 4). (**f**) Gross morphology of mice lung. H&E stained pathological tissue images (**g**) and lung injury scores (**e**) (*n* = 4). Original magnification × 200 and × 400. Bar = 50 μm. (**h**) Micro CT images of mouse lungs. (**i**) Lung volume quantification was analyzed by Analyze 12.0 software based on the Micro CT data (*n* = 3). Kit detected GSH (**j**) and MDA (**k**) contents in mouse lung tissue (*n* = 4). (**l**) qPCR was used to detect the expression of ferroptosis-related gene Ptgs2 mRNA (*n* = 4). Data were presented as the mean ± SD. ^⁎^*P* < 0.05 vs. Control group, ^#^*P* < 0.05 vs. SW group

As shown in Fig. 7a and b, DMF promoted the expression of Nrf2 in mice lung tissue. Immunofluorescence images also showed the same conditions as Western blots (Fig. 7c). Compared with the SW group, the SW + DMF group had a lower lung wet-to-dry ratio, indicating an improvement in pulmonary edema (Fig. 7d). Gross anatomy images showed that DMF treatment improves lung tissue hemorrhage and edema caused by seawater drowning (Fig. 7f). H&E slice staining images also showed that DMF treatment improved lung pathological damage in mice (Fig. 7e and g). As shown in Fig. 7h and i, Micro CT results showed that lung injury and deformation were improved in the SW + DMF group compared with the SW group, and the lung volume was also restored in the SW + DMF group. The results of GSH, MDA in lung tissue showed that compared with SW group, the content of GSH in SW + DMF group increased, and the content of MDA decreased (Fig. 7j and k). qPCR results showed that seawater caused an increase of *Ptgs2* mRNA expression in mouse lung, and DMF treatment reduced *Ptgs2* mRNA levels (Fig. 7l). These data indicate that DMF treatment inhibited ferroptosis and improved lung injury in mice caused by seawater drowning.

### Nrf2 deficiency aggravated lung injury and ferroptosis in mice induced by seawater drowning

**Fig. 8:**
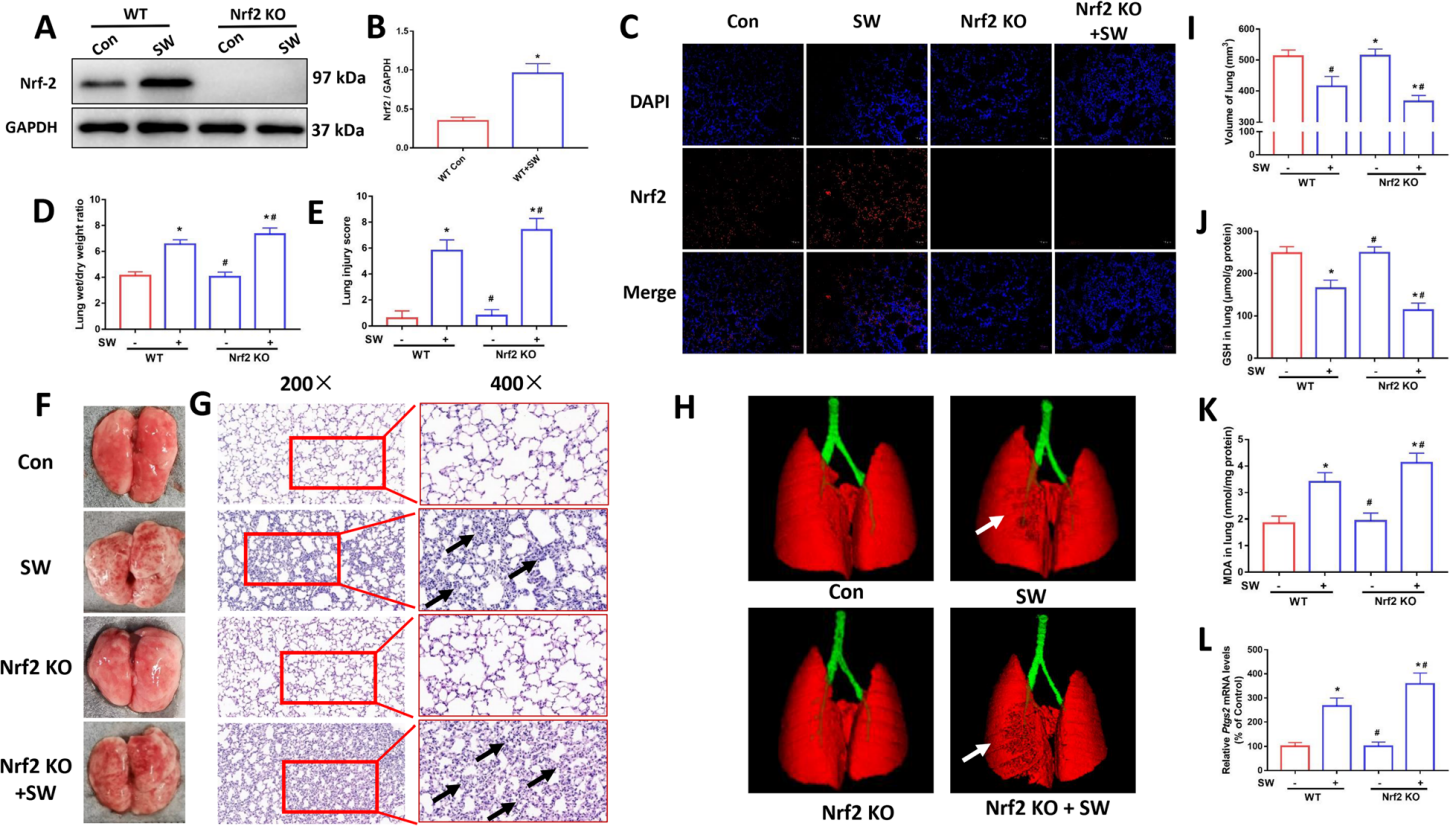
Nrf2 deficiency aggravated ALI and ferroptosis in mice induced by seawater drowning. Images (**a**) and quantification (**b**) of Nrf2 expression levels in lung were analyzed by Western blot (*n* = 3). (**c**) Immunofluorescence images were used to detect the expression of Nrf2 (red) in mouse lung tissue. Nuclei were counterstained with DAPI (blue). Original magnification × 200. Bar = 50 μm. (**d**) Lung wet/dry weight ratio (*n* = 4). (**f**) Gross morphology of mice lung. H&E stained pathological tissue images (**g**) and lung injury scores (**e**) (*n* = 4). Original magnification × 200 and × 400. Scale bars: 50 μm. (**h**) Micro CT images of mouse lungs. (**i**) Lung volume quantification was analyzed by Analyze 12.0 software based on the Micro CT data (*n* = 4). Kit detected GSH (**j**) and MDA (**k**) contents in mouse lung tissue (*n* = 4). (**l**) qPCR was used to detect the expression of ferroptosis-related gene Ptgs2 mRNA (*n* = 4). Data were presented as the mean ± SD. ^⁎^*P* < 0.05 vs. Control group, ^#^*P* < 0.05 vs. SW group

To confirm the protective effect of Nrf2 on drowning lung injury, we used Nrf2^−/−^ mice for further validation. As shown in Fig. 8a and b, we verified the knockout effect of Nrf2 in mouse lung tissue by Western blotting. The same result was obtained with the immunofluorescence image (Fig. 8c). Nrf2^−/−^ mice had a significantly higher lung wet-to-dry ratio (Fig. 8d), indicating more severe pulmonary edema. Gross anatomy and H&E staining images showed that seawater drowning caused more severe bleeding and pathological damage in Nrf2 knockout mice compared to wild-type mice (Fig. 8e-g). Similar results were obtained by Micro CT. The Nrf2 KO + SW group had the decreased lung volume than that in the SW group (Fig. 8h and i). Biochemical indicators also showed that the lower levels of GSH and the higher MDA in Nrf2^−/−^ mice compared with those in wild-type mice (Fig. 8j and k). In addition, the expression of *Ptgs2* mRNA in the Nrf2 KO + SW group was also higher than that in the SW group (Fig. 8l). These results indicated that Nrf2 deficiency aggravated ALI in mice induced by seawater drowning.

## Discussion

Drowning is one of the three major causes of unintentional injury death in the world [41]. Many people annually die mainly due to ALI or ARDS while no specific and effective treatments are currently available [5, 41]. Thus, it is very important to understand the key mechanisms and look for strategies to treatment seawater-induced ALI. It is generally recognized that many factors such as oxidation, inflammation, apoptosis and delayed cell proliferation be involved in the pathogenesis of seawater drowning-ALI [4]. In this study, we demonstrated that seawater exposure directly increased the levels of intracellular ROS and lipid ROS, and reduced GSH content and led to severe mitochondrial damage in MLE-12 cells and mice. Meanwhile, exposure to seawater add the content of MPO and MDA and decreases total superoxide dismutase activities. These results demonstrated that severe oxidative stress is the key factor in seawater drowning-induced ALI.

Ferroptosis is an iron-dependent, lipid peroxide-driven form of cell death. The new cellular death phenotype is mechanistically and phenotypically distinct from other cell death processes, e.g. apoptosis, autophagy, pyroptosis [15, 42]. However, whether and how ferroptosis plays a role in seawater drowning-induced ALI in mice never been explored. In this study, we firstly found that seawater exposure induced MLE-12 cell damage and death while inhibition of ferroptosis by ferrostatin-1 (Fer-1) improved cell viability and cell death. Furthermore, Fer-1 treatment reduced the levels of intracellular ROS and lipid ROS and MDA, and increased the levels of GSH and SOD. These results indicated that ferroptosis participated in MLE-12 cell damage caused by seawater exposure. The seawater-drowning mice also verified that ferroptosis-mediated lung injury. Gross anatomical morphology, HE slices staining and micro CT results consistently showed that inhibition of ferroptosis attenuated ALI in mice caused by seawater drowning.

Mitochondria are important organelles of oxidative metabolism and play a crucial role in ferroptosis [26]. Abnormal mitochondrial metabolism significantly contributes to rapid glutathione depletion and subsequent lipid ROS generation and ferroptosis [38]. In this study, we monitored mitochondria function using C11-BODIPY 581/591 and mitochondrial superoxide images by MitoSox Red fluorescent probe and DHE fluorescent probe and MMP assay. We found that seawater stimulation caused an increase in intracellular ROS, lipid ROS accumulation in MLE-12 cells. Furthermore, Fer-1 treatment reduced the levels of intracellular ROS and lipid ROS and MDA, and increased the levels of GSH and SOD in MLE-12 cells. Here, we presented evidences to demonstrate that the mitochondrion is indeed a crucial player in ferroptosis induced by seawater simulation.

**Fig. 9:**
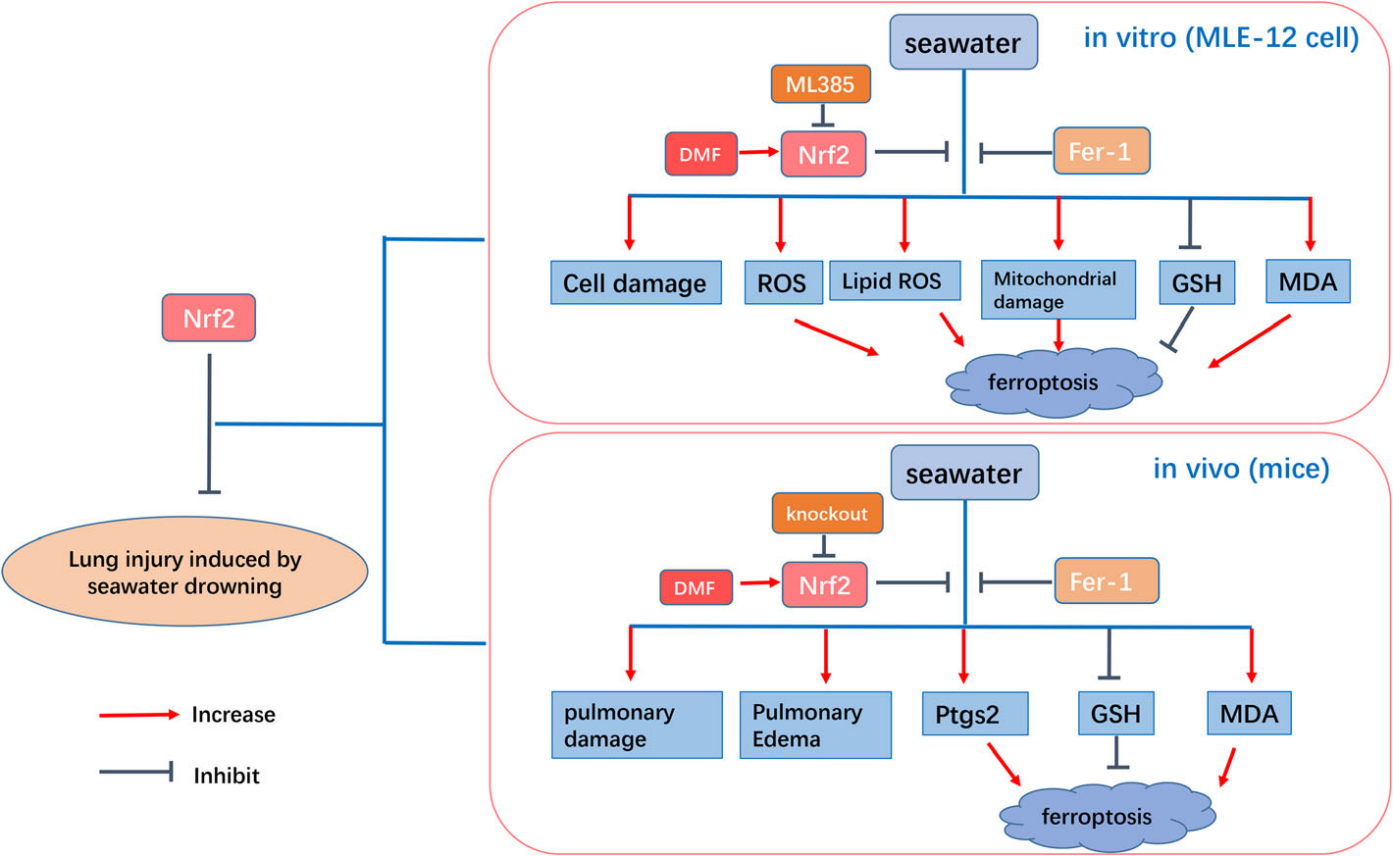
A schematic model for Nrf2 activation attenuating seawater drowning-induced ALI in vitro and in vivo

Ferroptosis has been involed in several pathophysiological conditions such as degenerative diseases, stroke, tumor suppression, and antiviral immunity. The mechanisms about ferroptosis are being hot topic in recent years [17, 27]. Elucidating how ferroptosis provokes ALI will expose new therapeutic opportunities to treat these diseases. Nrf2 is a core player in the regulation of antioxidant molecules in cells, regulating a variety of genes [11, 43]. Nrf2 activation has a beneficial effect on ischemia-reperfusion-induced lung injury [44], lipopolysaccharide-induced ALI [45] and ventilator-induced lung injury [46, 47]. Furthermore, Nrf2 inhibition promoted erastin or artesunate-induced ferroptosis through regulating redox homeostasis [43, 48]. In this study, we found that seawater exposure induced an increase in Nrf2 expression in MLE-12 cells. In addition, we used DMF to induce Nrf2 expression and translocation into the nucleus, which mitigated MLE-12 cell damage caused by seawater exposure. Furthermore, activation of Nrf2 pathway by DMF significantly attenuated GSH depletion and accumulation of MDA, ROS and lipid ROS induced by seawater exposure in MLE-12 cells. In addition, detection of mitochondrial membrane potential and mitochondrial ROS content also indicated that up-regulation of Nrf2 pathway improved mitochondrial function in MLE-12 cells. The results of qRT-PCR analysis also showed that Nrf2 inhibited *Ptgs2* mRNA expression, a key ferroptosis maker, in MLE-12 cells after seawater exposure. DMF treatment of wild-type mice also significantly ameliorated ALI induced by seawater drowning. The results of GSH, MDA and Ptgs2 mRNA also indicate that DMF-induced Nrf2 inhibited ferroptosis in the lungs of mice caused by seawater drowning. We further used Nrf2 knockout mice to verify the role of Nrf2 on ferroptosis in seawater drowned-ALI. As expected, Nrf2 knockout mice had more severe ALI and ferroptosis than those in wild-type mice after seawater drowning. These results demonstrated that Nrf2 inhibited ferroptosis and alleviated lung damage in mice caused by seawater drowning (Fig. 9).

There many important genes regulating ferroptosis process, such as *GPX4*, *Ptgs2* and *FTH1*. Ferroptosis process often led GSH depletion which involves in abnormal GSH synthesis cysteine supply controlled by system x_c_^−^, glutathione reductase and GPX4 [36]. GSH and GSH-associated metabolism provide the major line of defense for the protection of cells from oxidative and other forms of toxic stress. In this study, we found Nrf2 agonist DMF up-regulated *GPX4* mRNA expression. Meanwhile, we used the String database (protein-protein interaction) and found that Nrf2 and GPX4 did has positive relations. The p62–Keap1–Nrf2 antioxidant system may be responsible for the promoted function of Nrf2 on GPX4 expression [27]. Nrf2-mediated upregulation of the iron storage protein, ferritin, promoted cellular proliferation [40]. In this study, we found that Nrf2 activation increased *FTH1* mRNA expression. *Ptgs2*, also known as cyclooxygenase 2 (Cox-2), is considered to be a typical indicator of ferroptosis [42]. The network graphic showed that ferroptosis-related genes *FTH1*, *GPX4* and *Ptgs2* were associated with Nrf2, which molecularly explain the beneficent role in seawater drowning-induced oxidative stress damage.

This study still has several limitations. Firstly, although we found that DMF significantly increased the expression of Nrf2 and promoted the translocation of Nrf2 into the nucleus, DMF had Nrf2-independent role which directly induce adaptive and innate immune modulation [49] and has a direct effect on CD4 and CD8 T cells function [50] independent of Nrf2. Therefore, it is very necessary to use Nrf2 siRNA or Nrf2 KO mice with DMF treatment to exclude the off targeting effects of DMF. In addition, Ptgs2 and its downstream lipid metabolites (such as PGE2) contribute to acute lung injuries induced by ischemia/reperfusion or acid aspiration, therefore the definitive pathogenic role of Ptgs2 on seawater drowning-induced ALI remain to be determined. Further work on the mechanistic details of NrF2 on seawater drowning-induced ALI are required on the future work.

## Conclusions

Our in vivo and in vitro work firstly demonstrated that Nrf2 can inhibit ferroptosis and alleviate ALI induced by seawater drowning. These results elucidate a new mechanism underlying drowning-induced pulmonary damage and identify Nrf2 as a potential therapeutic target for the treatment of ALI.

## Data Availability

The datasets used and/or analyzed during the current study are available from the corresponding author on reasonable request.

## Abbreviations

Nrf2: Nuclear factor (erythroid-derived 2)-like 2
SW: Seawater
DMF: Dimethyl fumarate
ALI: Acute lung injury
ARDS: Acute respiratory distress syndrome
Fer-1: Ferrostatin-1
DHE: Dihydroethidium
ROS: Reactive oxygen species
SOD: Superoxide dismutase
GSH: Glutathione
MDA: Malondialdehyde
Ptgs2: Prostaglandin-endoperoxide synthase 2
FTH1: Ferritin heavy chain 1
GPX4: Glutathione peroxidase 4
MMP: Mitochondrial membrane potential
